# Biochemical and hemostatic description of a thrombin-like enzyme TLBro from *Bothrops roedingeri* snake venom

**DOI:** 10.3389/fchem.2023.1217329

**Published:** 2023-11-30

**Authors:** Augusto Vilca-Quispe, Aldo Alvarez-Risco, Mauricio Aurelio Gomes Heleno, Emilio Alberto Ponce-Fuentes, Corina Vera-Gonzales, Herly Fredy Enrique Zegarra-Aragon, Juan Luis Aquino-Puma, María Elena Talavera-Núñez, Shyla Del-Aguila-Arcentales, Jaime A. Yáñez, Luis Alberto Ponce-Soto

**Affiliations:** ^1^ Department of Biochemistry, Institute of Biology (IB), State University of Campinas (UNICAMP), Campinas, SP, Brazil; ^2^ Facultad de Administración y Negocios, Universidad Tecnológica del Perú, Lima, Perú; ^3^ Centro de Estudos de Venenos e Animais Peçonhentos (CEVAP), Universidade Estadual Paulista “Júlio de Mesquita Filho” (UNESP), Botucatu, SP, Brazil; ^4^ Laboratorio de Química de Proteínas, Universidad Católica de Santa María, Arequipa, Perú; ^5^ Departamento Académico de Química, Facultad de Ciencias Naturales y Formales, Universidad Nacional de San Agustín de Arequipa, Arequipa, Perú; ^6^ Facultad de Medicina, Universidad Nacional de San Agustín de Arequipa, Arequipa, Perú; ^7^ Escuela de Posgrado, Universidad San Ignacio de Loyola, Lima, Perú; ^8^ Facultad de Educación, Carrera de Educación y Gestión del Aprendizaje, Universidad Peruana de Ciencias Aplicadas, Lima, Perú

**Keywords:** serine protease, TLBro, Bothrops roedingeris, snake venom, thrombin-like enzyme, primary sequence

## Abstract

**Objective:** The current study’s objective is to characterize a new throm-bin-like enzyme called TLBro that was obtained from *Bothrops roedingeris* snake from a biochemical and hemostatic perspective.

**Methodology:** One chromatographic step was used to purify it, producing the serine protease TLBro. Molecular mass was estimated by SDS-PAGE to be between reduced and unreduced by 35 kDa. Tryptic peptide sequencing using Swiss Prot provided the complete amino acid sequence. Expasy.org by conducting a search that is limited to Crotalinae snake serine proteases and displaying a high degree of amino acid sequence.

**Results:** Ser (182) is inhibited by phenylmethylsulfonyl fluoride (PMSF), and TLBro demonstrated the presence of Asp (88) residues. It also deduced the positions of His (43) and Ser (182) in the set of three coordinated amino acids in serine proteases. It was discovered that this substrate had high specificity for BANA, Michaelis-Menten behavior with KM 0 point85 mM and Vmax 1 point89 nmoles -NA/L/min, and high stability between temperatures (15 to 70°C) and pHs (2 point0 to 10 point0). According to doses and incubation times, TLBro degraded fibrin preferentially on the B-chain; additionally, its activities were significantly diminished after preincubation with divalent ions (Zn_2_ and Cd_2_). When incubated with PMSF, a particular serine protease inhibitor, enzymatic activities and platelet aggregation were inhibited.

**Conclusion:** The findings revealed distinct structural and functional differences between the serine proteases, adding to the information and assisting in the improvement of the structure-function relationship.

## Introduction

Snakes are carnivorous animals that use their venom to immobilize and kill their prey. Snake venom is produced in venom glands located in the snake’s head, just behind the eyes. These glands are highly specialized structures that are different in size and shape among different types of snakes. Specifically, snake venom glands are composed of a collection of secretory cells called serous cells. The biochemistry of serous cells varies according to the snake species and its diet. Snake venoms contain different kind of components, including, neurotoxins, cytotoxins, proteolytic enzymes and coagulants, among others. These components are produced by serous cells and secreted into the venom through specialized ducts. The size of serous cells can vary from species to species, but they are usually around 10 μm in diameter. The serous cells are found in a complex glandular structure known as the venom gland. This gland is usually found on the head of the snake, near the hollow teeth that are used to inject venom into prey.

In 2017, WHO classified snakebite envenomation in category A of snakebite illness. This type of envenomation was reported to affect 2.7 million inhabitants annually, being mainly those residing in peripheral and poorly developed locations in tropical sites (Chippaux, 2017). Annually between 81,000 and 138,000 people die from snakebites which can cause paralysis, bleeding disorders and other damage (WHO, 2021). In Latin America, at least 70,000 cases of snakebites occur annually, although underreporting is suspected since they tend to occur in rural regions (Gutiérrez, 2014). In Peru, more than 2,000 cases of ophidism are reported annually, and snakes of the genus Bothrops are of greatest interest because they are responsible for most cases (Ministry of Health, 2020).

Taxonomically, Bothrops roedingeri is described as follows: Class: Sauropsida, Order: Squamata, Family: Viperidae. The manifestations of Bothrops poisoning are the result of the action of the different components of the venom (Warrell, 2010; Noutsos et al., 2022; Ralph et al., 2022), whose composition, as well as the biological properties of several species of Bothrops (Guerra-Duarte et al., 2015; Lomonte et al., 2020; Nina-Cueva et al., 2020; Amorim et al., 2023; Brás-Costa et al., 2023).

Snake venom serine proteases (SVSPs) are a fascinating and set of enzymes that have been the subject of intense study in recent years. These enzymes are found in snake venoms and are responsible for catalyzing different reactions within the coagulation cascade, the kal-likrein-kinin, fibrinolytic, and complement processes, as well as endothelial cells and blood platelets (Parry et al., 1998; Zelanis et al., 2015; Boldrini-França et al., 2020; Latinović et al., 2020; Megale et al., 2021; dos Santos et al., 2021; Vander dos Santos et al., 2018; Amorim et al., 2018). While each individual SVSP typically only catalyzes one or a few reactions within these processes, the high degree of mutual sequence identity among them belies their vast substrate specificity differences. Thrombin-like SVSPs (TL-SVSPs), one subset of SVSPs, are of particular interest to researchers. These proteinases are functionally related to thrombin, which plays a crucial role in blood clotting. TL-SVSPs are responsible for catalyzing a variety of reactions that are important for blood clotting, making them a key area of focus for researchers studying hemostasis. Despite the importance of TL-SVSPs, sequencing and structural studies of these enzymes have only occurred relatively recently. This is in part because snake venom research has historically been limited by the lack of venom and the difficulties inherent in working with these dangerous substances.

Among these enzymes, thrombin-like SVSPs (TL-SVSPs) are of particular interest due to their structural and functional similarity to thrombin. While TL-SVSPs have only recently been studied in depth, they hold great promise for the development of new treatments for a variety of medical conditions. As research in this area continues to progress, it is likely that we will continue to learn more about the fascinating world of SVSPs and their potential applications in medicine.

Based on the catalytic amino acid residues they contain, four different types of SVSPs have been identified: metalloproteinases (Modahl, Frietze, and Mackessy, 2018; Leonardi et al., 2019; Williams et al., 2019; Asega et al., 2020), serine (Carone et al., 2018; Belo et al., 2022; Morjen et al., 2022), aspartic (Teixeira et al., 2020), and cysteine (Suntravat et al., 2019; Tadokoro et al., 2020; Suntravat et al., 2021; Požek et al., 2022). Serine proteases are the most prevalent and extensively researched group of these. SVSPs, in accordance with Rawlings and Barrett (Barrett, Woessner, and Rawlings, 2012), are members of the clan PA subclan S and family S1. The capacity of TL-SVSPs to cleave fibrinogen is what distinguishes them (Stocker, Fischer, and Meier, 1982; Markland, 1998). Like thrombin, these enzymes cause cleavage of the Arg-Lys bonds of the fibrinogen chains, resulting in the conversion of fibrinogen to fibrin. Most TL-SVSPs cleave one of the chains, releasing either fibrinopeptide A (FPA) or fibrinopeptide B (FPB). TL-SVSPs are type A, B and AB (Pirkle, 1998) depending on this activity.

The Viperidae family is known for its venomous snakes, which contain a plethora of bioactive molecules with medicinal and toxic properties. One such class of molecules is serine proteases, specifically thrombin-like enzymes (TLEs), which are present in the SVSPs found in the Viperidae family. The catalytic triad (Ser195, His57 and Asp102) of these enzymes is highly conserved (Serrano and Maroun, 2005), enabling them to hydrolyze fibrinogen and liberate fibrinopeptides A and/or B, thereby promoting the formation of a weak fibrin clot (Magalhães et al., 2007).

Although TLEs have a thrombin-like action, they are not inhibited by heparin and do not activate blood coagulation factor XIII. This makes them a potential diagnostic or antithrombotic agent, or a valuable reagent for understanding the mechanisms of hemostasis. *In vitro*, these enzymes present coagulant and defibrinogenating activities, while *in vivo*, they display defibrin(ogen)ating properties. This report highlights the isolation, biochemical, and structural description of a TLE, TLBro, from the venom of *Bothrops roedingeri*, a species of venomous snake found in South America. The study sheds light on the properties of TLBro and its potential as a therapeutic agent. Serine proteases are enzymes that catalyze the cleavage of peptide bonds using a serine residue at their active site. They are involved in a plethora of biological processes. SVSPs, which belong to the serine protease family, have attracted attention due to their ability to cause pathological effects in humans and animals, including hemorrhage, tissue damage, and paralysis.

The fibrinolytic system is a crucial mechanism for maintaining hemostasis. However, disturbances in this system can lead to thrombosis, a pathological condition characterized by the formation of blood clots that obstruct blood flow. TLEs are relevant in the fibrinolytic system by hydrolyzing fibrinogen and releasing fibrinopeptides A and/or B, which promote the formation of a weak fibrin clot. TLBro, the TLE isolated in this study, has been observed to have defibrinogenating activity (Stocker, 1990; Pérez et al., 2008), suggesting that it can be used as a diagnostic or antithrombotic agent. In addition, it is *in vitro* coagulant activity makes it a valuable reagent for elucidating the mechanisms of hemostasis (Rojnuckarin et al., 2006; Calvete, 2010). This study also presents the three-dimensional structure of TLBro, which allow understanding the mechanism of action of TLEs.

## Materials and methods

### Materials

*Bothrops roedingeri* venom was collected and provided by Dr. Corina Vera Gonzales. The analytical or sequencing-grade chemicals and reagents used in this work were all acquired from Sigma Chemical Co. in St. Louis, Missouri, United States. Analytical or sequencing-grade reagents were used in this experiment. The source of animals used in current study was Animal Services Unit of UNICAMP.

### Reverse phase HPLC

Reverse-phase HPLC was used to purify TLBro from *Bothrops roedingeri* according to the method described by Vilca-Quispe et al. (2010). Briefly, 5 mg of total venom was dissolved in 200 µL buffer A (0.1% trifluoroacetic acid-TFA) and centrifuged at 4,500 rpm for 10 min. The supernatant was then subjected to reverse-phase HPLC using a Waters PDA 991 system, and a Discovery^®^ BIO Wide Pore C5 analytical column (25 cm × 4.6 mm) (Supelco, Sigma-Aldrich Co., United States) was pre-equilibrated with buffer A for 10 min. The protein was eluted using a linear gradient of buffer B (66.5% acetonitrile in buffer A), and the chromatographic cycle was monitored at 280 nm (flow rate of 1 mL/min). Elution was followed by storage and lyophilization at −20°C for the fraction.

### Calculate of kinetic indicators

The synthetic substrate N-benzoyl-L-arginine-ρ-nitroanilide (BaρNA), modified for 96-well ELISA plates, was used to measure the thrombin enzyme activity. In a final volume of 270 μL, the standard assay mixture consisted of 50 µL of buffer (100 mM NaCl, 10 mM Tris-HCl, 10 mM CaCl_2_, pH 8.0), 16 µL of water, 200 µL of a substrate, and 4 µL of the thrombin-like enzyme (TLBro). The mixture was incubated for up to 40 min at 37°C, with absorbance readings every 10 min after adding TLBro enzyme (4 µg). The enzyme’s reaction onset velocity (Vo) was determined based on the amount of p-nitroaniline released (Erlanger, Kokowsky, and Cohen, 1961). TLBro was incubated at different substrate concentrations, and the reaction for each concentration was repeated three times. The Michaelis constant (KM) and maximum velocity (Vmax) were estimated using the Michaelis-Menten and Lineweaver-Burk equations (double reciprocal plot). The effect of temperature on the enzyme was established by incubating the enzyme at different temperatures of 15°C, 25°C, 30°C, 35°C, 40°C, 45°C, 55°C, and 70°C and evaluating the enzyme activity. Similarly, the optimum pH for TLBro (serine protease) was determined by incubating the enzyme in buffers of different pH (100 mM sodium citrate, pH 3 and 4; 100 mM sodium acetate, pH 4.5 and 5.5, 100 mM sodium phosphate, pH 6 and 7; 100 Mm Tris-HCl, pH 7.5, 8.5; and 100 mM NaOH glycine, pH 9 and 10), followed by an enzyme activity assay.

### Fibrinogen-clotting activity

Clotting times were estimated using the technique described by Vilca-Quispe et al. (2010). A unit of fibrinogen clotting activity was defined as the amount of enzyme required to clot a fibrinogen solution in 1 min. Coagulation was represented by the coagulation index (CI).

### Fibrinogenolytic activity

The fibrinogenolytic activity was estimated by incubating 20 µL (1:1) of TLBro—4 with 0.9 mL of bovine fibrinogen (0.2%) in 10 mM Tris-HCl (pH 7.4) at 37°C (Gao et al., 1998). Consecutively, 0.9 mL of denaturing solution [0.05 mM Tris-HCl; pH 6.5; 10 mM urea; 10% β-mercaptoethanol; 2% SDS; and bromophenol blue (0.05%, w/v)] was added to complete the reaction at different times (0.5, 1, 2 and 6 h), and to examine fibrinogen degradation products were taken aliquots. SDS-PAGE was used to evaluate samples under reducing conditions by employing a 12.5% polyacrylamide gel, and protein staining was performed with Coomassie Brilliant Blue R-250.

### Platelet aggregation

This test was performed as described by Born and Cross (1963). Platelet-rich plasma (PRP) was obtained from immediate centrifugation at 290 rpm for 15 min at room temperature of blood anticoagulated with Alsever’s solution (1 vol of anticoagulant per 5 vol of blood), and platelet-poor plasma (PPP) was obtained from centrifugation at 1,500 rpm for 10 min at room temperature of the remaining cell suspension. In a turbidimetric assay using the whole blood platelet aggregometer PA-04 (Qualiterm Eletrônica, Brazil), platelet aggregation was recorded for 5 min. In the assays, 400 µL of PRP was used in siliconized glass cuvettes at 37°C under stirring, and after a 5 min preincubation, platelet aggregation was triggered with aliquots of the purified enzyme. In the control experiment, platelet aggregation was induced with ristocetin (5 mcg/mL), and different doses of TLBro (10 and 20 µg/500 µL) were tested for platelet aggregation.

### Inhibitor studies

The goal of the study was to determine how different protease inhibitors affected the activity of enzymes that had been purified on BApNA. Newly made stock solutions of the inhibitors were used. While *Didelphis marsupialis* serum, soybean trypsin inhibitor type I, and ethylenediaminetetraacetic acid (EDTA) were dissolved according to Valeriano-Zapana et al. (2012). Utilizing a VERSAMAX 190 microplate reader, the assays were run following the 3 times, and the absorbance at 410 nm was estimated.

### Amino acid analysis

The amino acid composition of the purified TLBro sample was determined using a Pico-Tag analyzer (Waters Systems) (Heinrikson and Meredith, 1984). For this purpose, it was used the technique described by Silva et al. (2001).

### Mass spectrometry

A hybrid quadrupole-time-of-flight (Q-TOF) mass spectrometer (Q-TOF Ultima, Mi-cromass, Manchester, United Kingdom) equipped with a nanospray source operating in positive ion mode was used to obtain all mass spectra. The ionization conditions had a collision energy of 10 V, a capillary voltage of 2.3VkV, a cone voltage of 30 V, and a 100 V RF1 lens. The source temperature was 70°C, and the cone gas was N2 at a flow rate of 80 L/h; no nebulizer gas was used to acquire the sprays. Argon was used for collisional cooling and ion fragmentation in the collision cell, and external calibration was performed using sodium iodide over the mass range of 50–3,000 m/z. All spectra were obtained using a TOF analyzer at “Vmode” (TOF kV = 9.1), and the MCP voltage was set to 2,150 V.

### *De novo* sequencing of triptic peptides

Before injecting the alkylated tryptic peptides separated by RP-HPLC into the mass spectrometer source at a flow rate of 500 nL/min, they were lyophilized and resuspended in 20% acetonitrile in 0.1% TFA. To identify the ion of interest, an ESI/MS mass spectrum (TOF-MS mode) was obtained for each HPLC fraction spanning a mass range of 400–2,000 m/z, which was then fragmented in the collision cell (TOF MS/MS mode). Distinct collision energies were applied depending on the mass and charge states of the ions. Production spectra were obtained using a TOF analyzer and deconvoluted using the MassLynx-MaxEnt 3 algorithm. The PepSeq program provided by MassLynx was used to analyze the singly charged spectra manually.

### Analysis of tryptic digests

Before adding trypsin (Promega-Sequence Grade Modified), the protein was reduced (DTT 5 mM for 25 min at 56°C) and alkylated (iodoacetamide, 14 mM for 30 min). The sample was incubated for 16 h at 37°C after treatment with trypsin (20 ng/L in 0.05 M NH_4_HCO_3_), and 0.4% formic acid was added to halt the reaction. The sample was centrifuged at 2,500 rpm for 10 min, the supernatant was dried in a speed vacuum, and the pellet was discarded. RP-UPLC C18 (100 μm × 100 mm) (NanoAcquity UPLC, Waters) coupled with nano-electrospray tandem mass spectrometry on a Q-Tof Ultima API mass spectrometer (MicroMass/Waters) at a flow rate of 600 nL/min was used to obtain the peptides separately. To identify the ion of interest, an ESI/MS mass spectrum (TOF MS mode) was obtained for each HPLC fraction in the 100–2,000 m/z mass range prior to performing a tandem mass spectrum; therefore, these ions were fragmented in the collision cell (TOF MS/MS mode). The RP-UPLC C18 (100 μm × 100 mm) (nanoAcquity UPLC, Waters) coupled to nano-electrospray tandem mass spectrometry on a Q-Tof Ultima API mass spectrometer (MicroMass/Waters) at a flow rate of 600 nL/min used to obtain the peptides separately. To identify the ion of interest, an ESI/MS mass spectrum (TOF MS mode) was achieved for each HPLC fraction in the 100–2,000 m/z mass range previous to performing a tandem mass spectrum; thereon, these ions were fragmented in the collision cell (TOF MS/MS mode). The Masslynx 4.1 software package (WATERS) was used to process the raw data files performed by LC-MS/MS; they were analyzed utilizing the MASCOT search engine version 2.3 (Matrix Science Ltd.) against the snake database employing the following parameters: fragment mass tolerance of ±0.1 Da, peptide mass tolerance of ±0.1 Da, oxidation in enzymes such as modifications in methionine and trypsin.

### Statistical analysis

The data is presented as the average value with the standard error of the mean to reflect the uncertainty in the estimate. To determine whether there are significant differences between the experimental groups and the control group, an analysis of variance (ANOVA) was conducted, and *post hoc* Dunnett’s test was employed when multiple groups were compared to the control group. A *p*-value less than 0.05 was considered statistically significant, implying that the observed differences were not likely due to chance.

## Results

### Isolation and biochemical characterization

Fractionation of *Bothrops roedingeri* venom by RP-HPLC on a Discovery^®^ BIO Wide Pore C5 analytic column resulted in twenty peaks (1–21).

**FIGURE 1:**
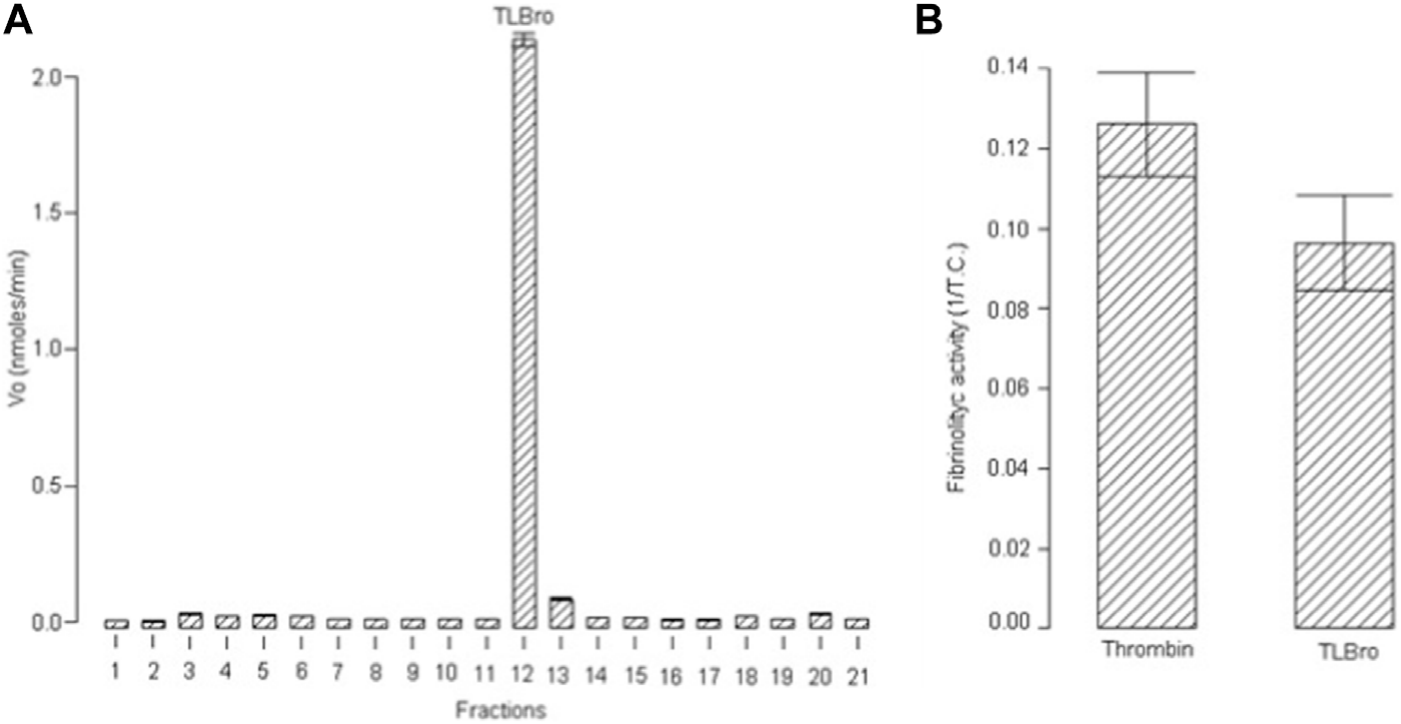
**(A)** Proteolytic activity of fraction 12 (TLBro) RP-HPLC serine protease. **(B)** The fibrinogen-clotting activity was determined by the inverse of coagulation time to the first sign of fibrin net formation.

**FIGURE 2:**
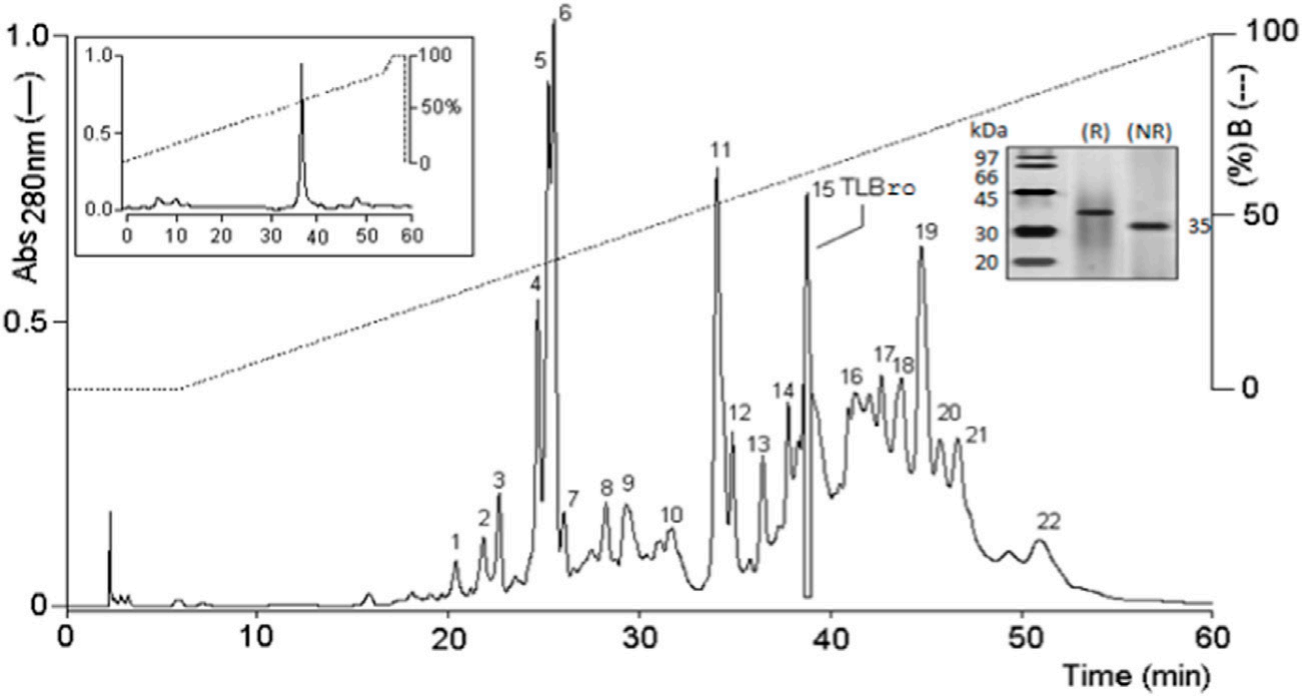
RP-HPLC on C5 chromatography of Bothrops roedingeri venom. The main fractions obtained were identified as 1 at 22. Fraction 15 corresponds to a serine protease with an activity similar to thrombin. Re-purification of serine protease, TLBro are shown at the top of the figure.

Peak 15, also known as TLBro, was identified and the eluted fraction was analyzed for coagulant, fibrinogenolytic, and proteolytic activities. The results showed that Peak 15 exhibited proteolytic activities (as shown in Figure 1A) and a significant coagulant effect (as shown in Figure 1B). The purity of this peak was confirmed by re-chromatography on an analytical RP-HPLC Discovery^®^ BIO Wide Pore C5 analytic column, which revealed only one peak. Furthermore, SDS-PAGE analysis demonstrated the presence of one electrophoretic band with a molecular weight of approximately 35 kDa (as shown in Figure 2), both in the presence and absence of DTT (1M).

**FIGURE 3:**
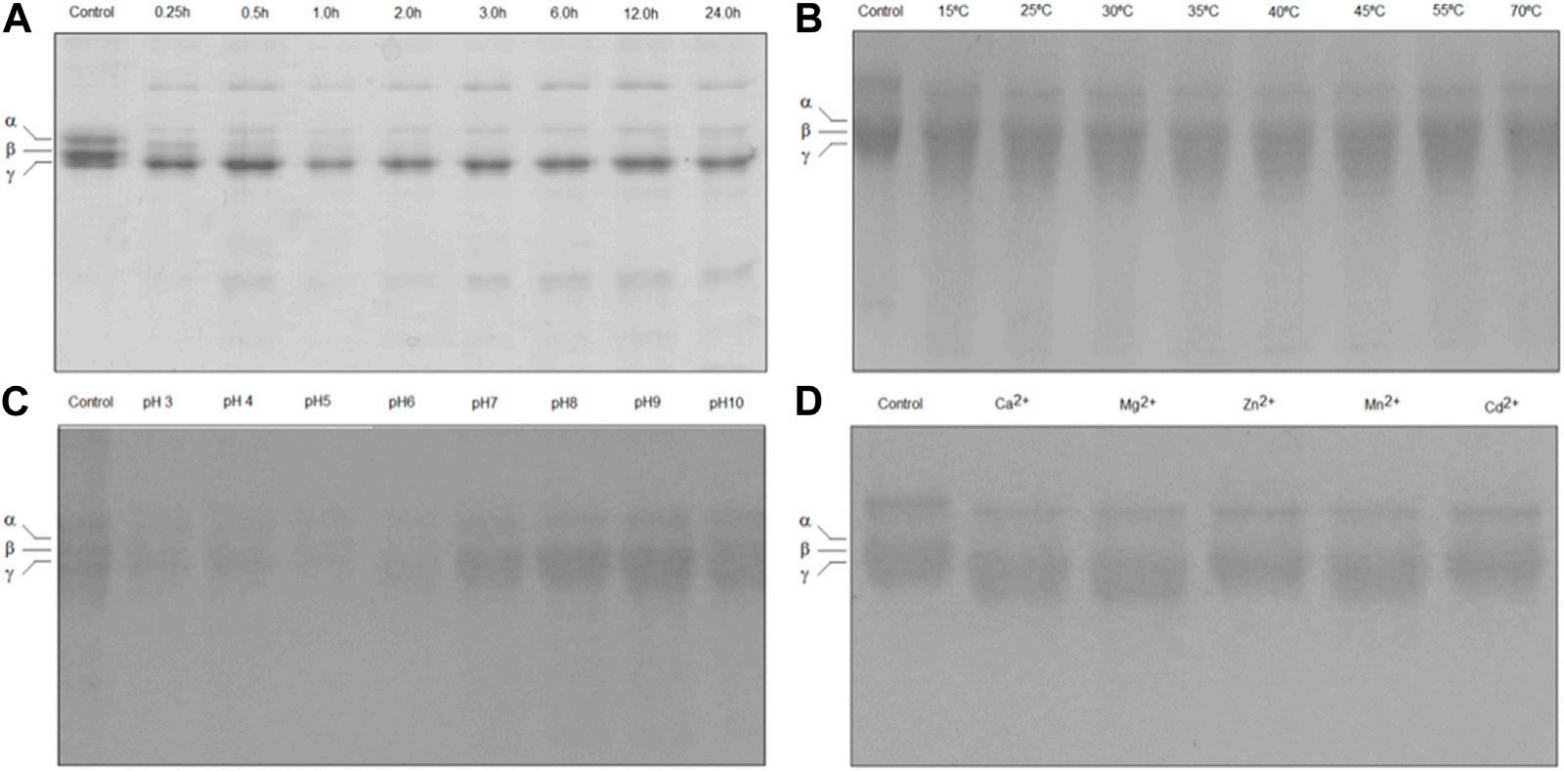
Fibrinogen-clotting assay. **(A)** The degradation analysis of bovine fibrinogen subjected to TLBro activity was conducted at various incubation times (0.5, 1, 2, 3, 6, 9, 12, and 24 h) at 37°C. The outcome revealed the fibrinogen molecule’s initial state at 0 min, exhibiting the presence of α, β, and γ chains. The experiment disclosed that the α and β chains of the fibrinogen molecule underwent clotting. All experiments were performed in triplicate, and the results are expressed as the mean ± S.E, with a significance level of *p* < 0.05. **(B)** The enzyme (20 μg) was incubated at different temperatures (15°C, 25°C, 30°C, 35°C, 40°C, 45°C, 55°C and 70°C) for 60 min, pH 7.5. **(C)** The enzyme (20 μg) was incubated at the different pH values (3.0, 4.0, 5.0, 6.0, 7.0, 8.0, 9.0 and 10) for 60 min at 37°C. **(D)** The TLBro (20 μg) incubated in the presence of metal ions (Ca^2+^, Mg^2+^, Zn^2+^; Mn^2+^ and Cd^2+^) each metal ion’s final concentration: 0.05 M) for 60 min at 37°C, pH 7.5.

The ability of TLBro to induce clotting of bovine fibrinogen was demonstrated by a fibrinogen-clotting assay. SDS-PAGE analysis of the reaction mixtures containing various concentrations of TLBro revealed that 20 μg of TLBro hydrolyzed the Aα chain of fibrinogen almost completely and partially cleaved the Bβ chain after 2 h of incubation, as illustrated in Figure 3A. Notably, no significant changes in the electrophoretic mobility of the γ chain were observed even after prolonged incubation (24 h). These findings suggest that TLBro selectively cleaves the Aα and Bβ chains of bovine fibrinogen, releasing FPA and FPB, and preferentially acts upon the Bβ chain while sparing the γ chain. The optimal temperature and pH range for TLBro to degrade bovine fibrinogen were found to be 25°C–45°C and 4.5–9.5, respectively, as demonstrated in Figures 3B, C. In addition, metal ion assays showed that Zn^2+^ and Cd^2+^ inhibited the fibrinogenolytic activity of TLBro, while Ca^2+^, Mg^2+^, and Mn^2+^ did not, as shown in Figure 3D.

### Composition amino acid

The amino acid analysis conducted on TLBro disclosed its intricate composition, indicating the presence of D/33, T/28, S/27, E/21, P/24, G/33, A/22, C/12, V/18, M/4, I/20, L/25, Y/10, F/7, K/12, H/11 and R/14 residues at different percentages. Remarkably, TLBro exhibited a conspicuous abundance of D, G, T, and S amino acids, which are characteristic residues of acidic proteins.

### De novo sequencing of protein

To obtain comprehensive insights into the structural features of the native protein, it underwent alkylation and digestion prior to ESI-MS/MS analysis. The ESI-MS/MS approach facilitated the deduction of sequences, which yielded nine peptides of alkylated TLBro (as presented in Table 1). RP-HPLC was employed to fractionate the alkylated protein digest into several chromatographic peaks, each of which was manually collected and lyophilized. The ESI-MS/MS method was applied to *de novo* sequence each peptide peak.

**TABLE 1:** The peptides were separated before being subjected to sequencing using mass spectrometry. From the alkylated tryptic peptides, the MS/MS-derived sequence was obtained.

TLBro			
TLBro HPLC fraction	Measurement of mass (Da)	Sequence of amino acid	Theoretical mass (Da)
1	1511.6787	VI/LGGDECNI/LNEHR	1511.5957
2	1068.5968	FL/IVAL/IYTSR	1068.5952
3	1419.7002	SL/IMNI/LYLGMHNK	1419.7044
4	964.4362	FDDEQ/KRR	964.4110
5	753.4609	L/INRPVR	753.4270
6	1188.6325	WDK/QDI/LMLI/LR	1188.6304
7	1189.6165	I/LMGWGTI/LSPTK	1189.6163
8	1060.5587	TL/ICAGI/LLEGGK	1059.5728
9	1167.5560	VSDYTEWI/LR	1167.5544

**FIGURE 4:**
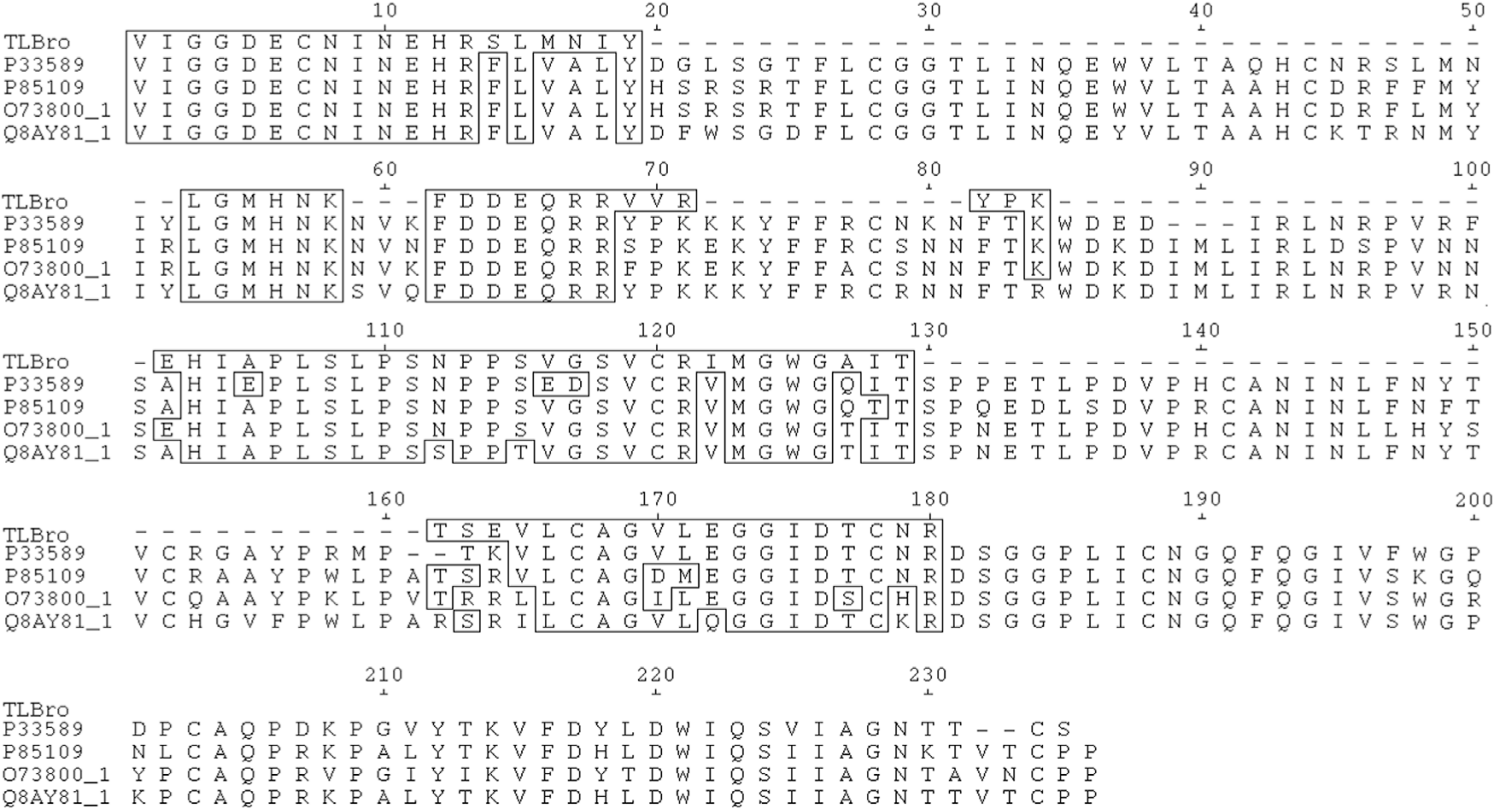
The TLBro amino acid sequence was aligned with selected sequences of serine proteases acquired from the SwissProt database (http://br.expasy.org), including P33589 from *Lachesis muta muta* (Magalhaes et al., 2007), O73800_1 from *Gloydius halys* (Jeong et al., 2001), P85109 from *Gloydius brevicaudatus* (Sakai et al., 2006), and Q8AY81_1 from *Viridovipera stejnegeri* (Zhang et al., 1998). Undetermined amino acid residues were denoted by (-), while identical residues were highlighted in boxes. The acidic amino acid residues of the catalytic triad were marked with an asterisk (*).

The primary structure of TLBro was determined by digesting the sequence with trypsin and deducting it from the SwissProt database (Figure 4). TLBro presented a sequence of 87 amino acid residues sequenced, being TLBro: VIGGDECNINEHRFLVALYT SRS------------------------LMNIYLGMHNK---FDDEQRRLNRP----------VRWDKDIMLIR----------------------------IMGWGTIS------------------------------PT—KVLCAGVLEGGIDTCNRT----L-CAGILEG----G--------------KVSDYTEWIR.

From TLBro, nine peptides with molecular masses of 1511.6787 Da (peak 1), 1068.5968 Da (peak 2), 1419.7002 Da (peak 3), 964.4362 Da (peak 4), 753.4609 Da (Peak 5) 1188.6325 Da (peak 6), 1189.6165 Da (peak 7), 1060.5587 Da (peak 8) and 1167.5560 Da (peak 9). After determining these molecular masses and utilizing iodoacetamide, the cysteines presented in the peptides were alkylated (Figure 4).

**FIGURE 5:**
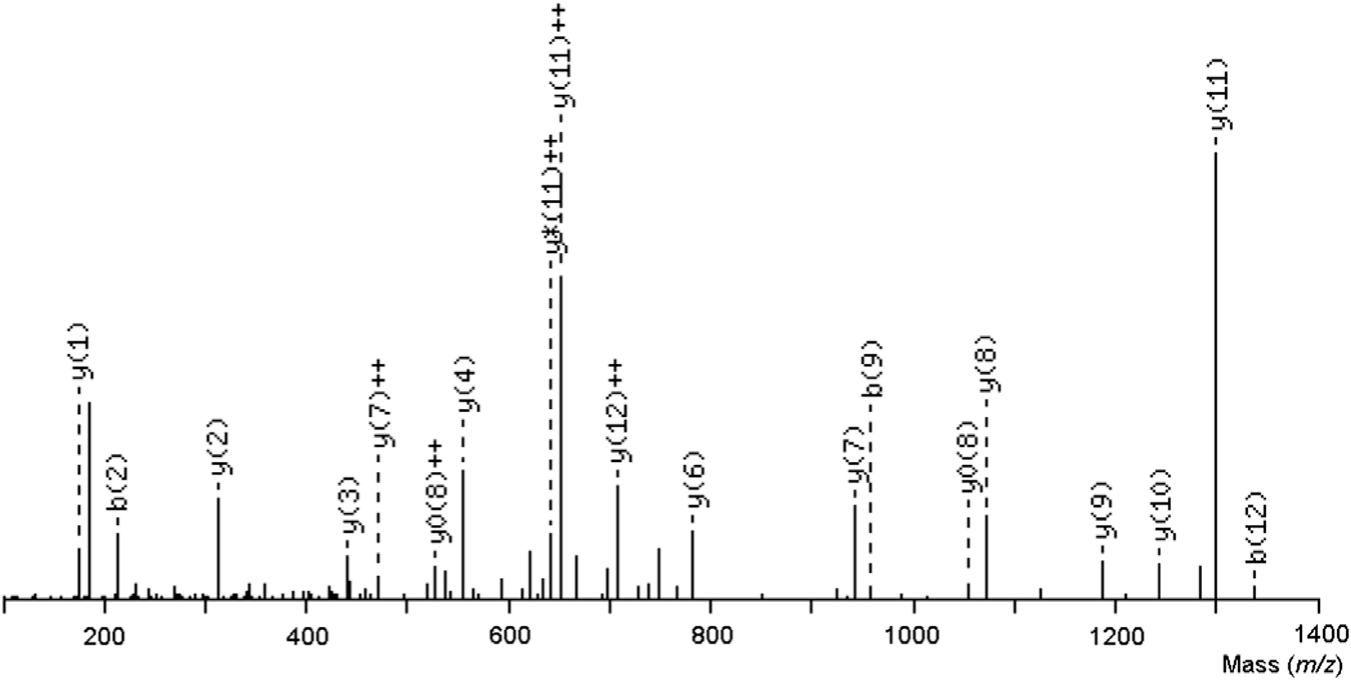
An ion series specific to the central sequence, the y-ion series, as well as a minor series of b-ions that complement it were both visible in the tryptic ion’s MS/MS spectrum, which had a charge of two and a mass-to-charge ratio of 1454.6572. The sequence of the TLBro tag, VIGGDECNINEHR, was inferred from this ion series.

The VI/LGGDECNI/LNEHR peptide was eluted in fraction 1 of TLBro, and its sequence was determined via tandem MS spectra, as illustrated in Figure 5. The deduced sequence of TLBro protein displayed a high degree of homology with the sequence of a serine protease from venomous snakes, as identified via Blast-p database search. The primary sequence of the serine protease TLBro, a thrombin-like protein from Bothrops roedingeri, was deduced, and it showed the presence of His, Asp, and Ser residues in the catalytic triad positions. The region surrounding the catalytic site of TLBro shares a high degree of similarity with other serine proteases, according to a comparison of its sequence with other thrombin-like proteins from snake venom (Figure 5).

### Evaluation of enzyme activity of thrombin like TLBro

**FIGURE 6:**
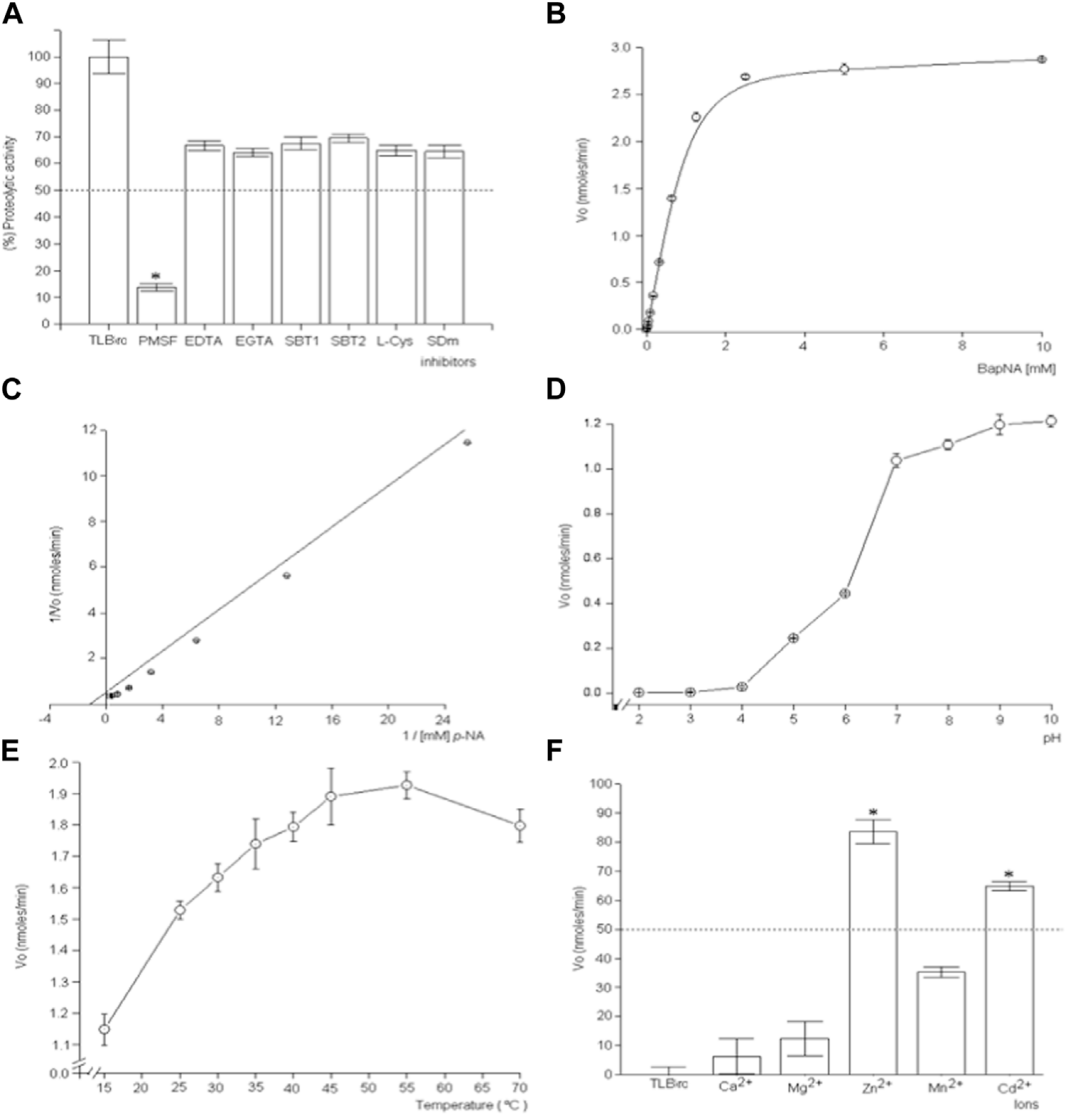
The investigation focuses on the kinetic characteristics and inhibitory effects of TLBro, a thrombin-like enzyme found in *Bothrops roedingeri* snake venom. The study encompasses a diverse range of experimental approaches, including **(A)** the use of inhibitors such as soybean trypsin inhibitor EDTA, SBTI, and PMSF to hinder TLBro’s proteolytic activity, **(B)** the examination of Michaelis-Menten curves to gauge the impact of substrate concentration on TLBro kinetics, **(C)** the construction of a Lineweaver-Burk plot to analyze the reciprocal relationship between substrate concentration and TLBro velocity, **(D)** the evaluation of the influence of pH on TLBro activity, **(E)** the assessment of the effects of temperature on TLBro proteolytic activity, and **(F)** the incubation of TLBro with various metal ions, including Ca^2+^, Mg^2+^, Zn^2+^, Mn^2+^, and Cd^2+^. Positive control thrombin-like activity of TLBan from *Bothrops andianus* snake venom **(A)**.

TLBro was found to possess proteolytic activity towards DL-BAρNA, as indicated by the cleavage of this substrate. The enzymatic activity of TLBro was inhibited by PMSF, a serine protease inhibitor, while EDTA and SBTI had a weaker inhibitory effect. In fact, the inhibitory effect of PMSF was concentration-dependent, with complete inhibition of TLBro activity observed at a concentration of 5 mM. On the other hand, EDTA did not inhibit the fibrinogenolytic activity of TLBro within a 60-min incubation, suggesting that TLBro is a serine protease rather than a metalloprotease (Figure 6).

Further characterization of TLBro revealed that it exhibited classic Michaelis-Menten behavior, with KM and Vmax values of 0.85 mM and 1.89 nmoles ρ-NA/lt/min/mg, respectively. The optimum pH for TLBro activity was 9.0, while the maximum enzyme activity was observed at 55°C. Metal ion assays showed that TLBro activity was inhibited by Zn^2+^ and Cd^2+^, but not by Ca^2+^, Mg^2+^, and Mn^2+^. Overall, these results provide important insights into the biochemical properties of TLBro and its potential biological functions.

**FIGURE 7:**
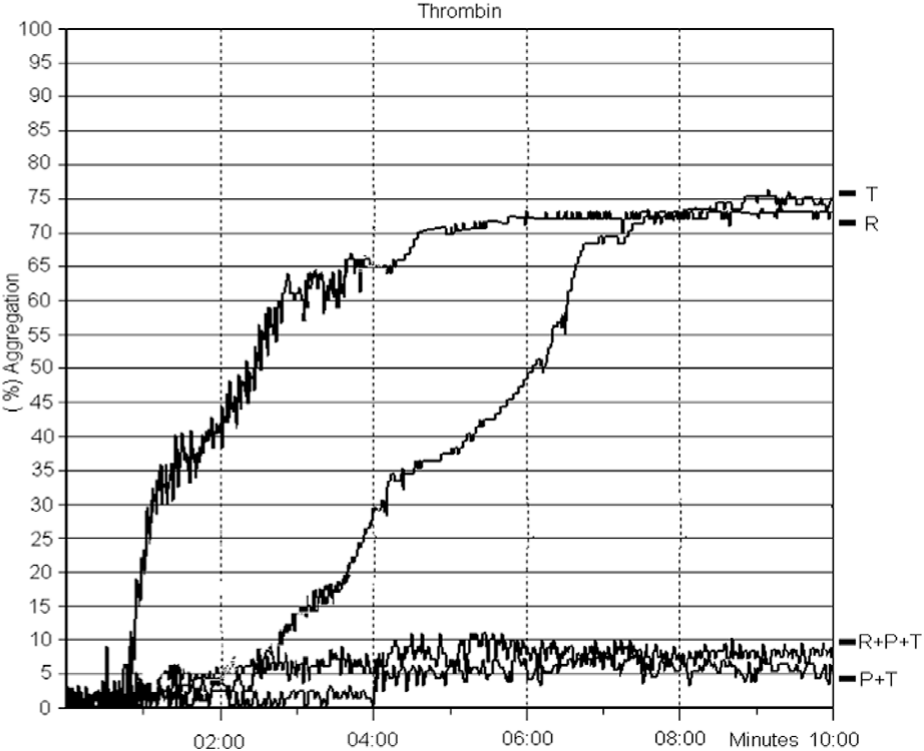
The introduction of TLBro led to platelet aggregation. PRP was subjected to a 10-min incubation period with TLBro (5 µg) under constant stirring at 37°C. Simultaneously, light transmittance was recorded to monitor the changes in the sample.

At concentrations of 5 g/mL (1:1, w/v) of TLBro when incubated with platelet-rich plasma (PRP) isolated from anticoagulated rat blood, TLBro stimulated robust aggregation of platelet (as shown in Figure 7).

## Discussion

Hemostasis and thrombosis researchers have long been fascinated by the influence of snake venom components on blood coagulation disturbances, with a wealth of literature attesting to their practical and theoretical value in laboratory investigations and clinical studies (Kini, 2005). This study presents a highly effective and comparatively simple protocol for isolating TLBro, a thrombin-like serine proteinase of exceptional purity from *Bothrops roedingeri* venom. Under reducing conditions, the single polypeptide chain of TLBro exhibits a molecular weight of about 35 kDa. TLBro’s micro-heterogeneity (Calvete, 2010) is caused by its carbohydrate content (Kini, 2005; Serrano, 2013), in contrast to other thrombin-like enzymes isolated from snake venom, which are single-chain proteins with molecular weights ranging from 26 to 67 kDa.

TLBro’s primary structure was determined through the SwissProt database’s deduced sequencing method and aligned with other homologous snake venom thrombin sequences. This alignment revealed that TLBro was quite similar to other serine proteases, as demonstrated in Figure 4. TLBro also exhibited high sequence homology with other snake venom thrombin-like enzymes, including P33589 from *Lachesis muta muta*, O73800_1 from *Gloydius halys* (Jeong, Yoo, and Kim, 2001), P85109 from *Gloydius brevicaudatus* (Sakai et al., 2006), and Q8AY81_1 from *Viridovipera stejnegeri* (Zhang et al., 1998). The pairing of each Cys residue in these enzymes is the same as in mammalian trypsin, and all Cys residues are disulfide bonded (Markland, 1998; Kini, 2005; Ponce-Soto et al., 2007; Vilca-Quispe et al., 2010; Serrano, 2013).

As the first N-terminal residue, valine is shared by the majority of snake venom thrombin, supporting the high homology of coagulant enzymes (Tang et al., 2009). *In vitro*, TLBro showed high clotting and fibrinogenolytic activities as well as esterase activity up-on DL-BAPNA. In a wide range of temperatures (25°C–45°C) and pH levels (4.5–9.5), TLBro demonstrated adequate cleavage activities on bovine fibrinogen. Similar mammalian serine proteases have been found to be inhibited by PMSF and -mercaptoethanol, while EDTA serves as a chelating agent for divalent metal ions. The addition of PMSF and -mercaptoethanol, but not EDTA, inhibited TLBro’s enzyme activity, demonstrating that it is not a metalloprotease but rather a thermally stable serine protease, similar to the majority of fibrinogen-clotting enzymes in snake venoms (Stocker, Fischer, and Meier, 1982; Markland, 1998; Lewis and Garcia, 2003), According to most other serine proteases (Swenson and Markland, 2005; Ponce-Soto et al., 2007; Santana, LarraÑaga, and Lozano, 2008; Vilca-Quispe et al., 2010), TLBro displays the typical characteristics of snake venom serine proteases.

The majority of thrombin enzymes found in snake venom are active on a variety of organic and synthetic substrates, and specific inhibitors like PMSF, leupeptin, aprotinin, and benzamidine frequently alter these enzymes’ characteristics (Serrano and Maroun, 2005). The fact that protease inhibitors were susceptible to TLBro when fibrinogen was used as a substrate showed that PMSF reduced the enzyme’s catalytic activity, most likely by altering the active serine residue. By encouraging platelet aggregation and release reaction, platelet-aggregating enzymes have an impact on platelet-rich plasma or washed platelet suspensions. At nanomolar concentrations, these proteins, which are essential, facilitate their activities on platelets by limiting proteolysis (Kini, 2005). The thrombin-like activity of TLBro, a serine protease, is similar to that of proteins that act similarly to thrombin and causes platelet aggregation in both PRP and washed platelet suspensions (Figure 6B).

## Conclusion

TLBro can be categorized as a novel thrombin-like enzyme derived from the venom of the *Bothrops roedingeri* snake based on its biochemical, enzymatic, and pharmacological properties. This serine protease could act as a molecular model, directly or indirectly, for clinical applications, similar to other thrombin-like enzymes. Its eventual use in the treatment and prevention of cardiovascular diseases and strokes may result from this.

## Data Availability

The original contributions presented in the study are included in the article/Supplementary Material, further inquiries can be directed to the corresponding author.
